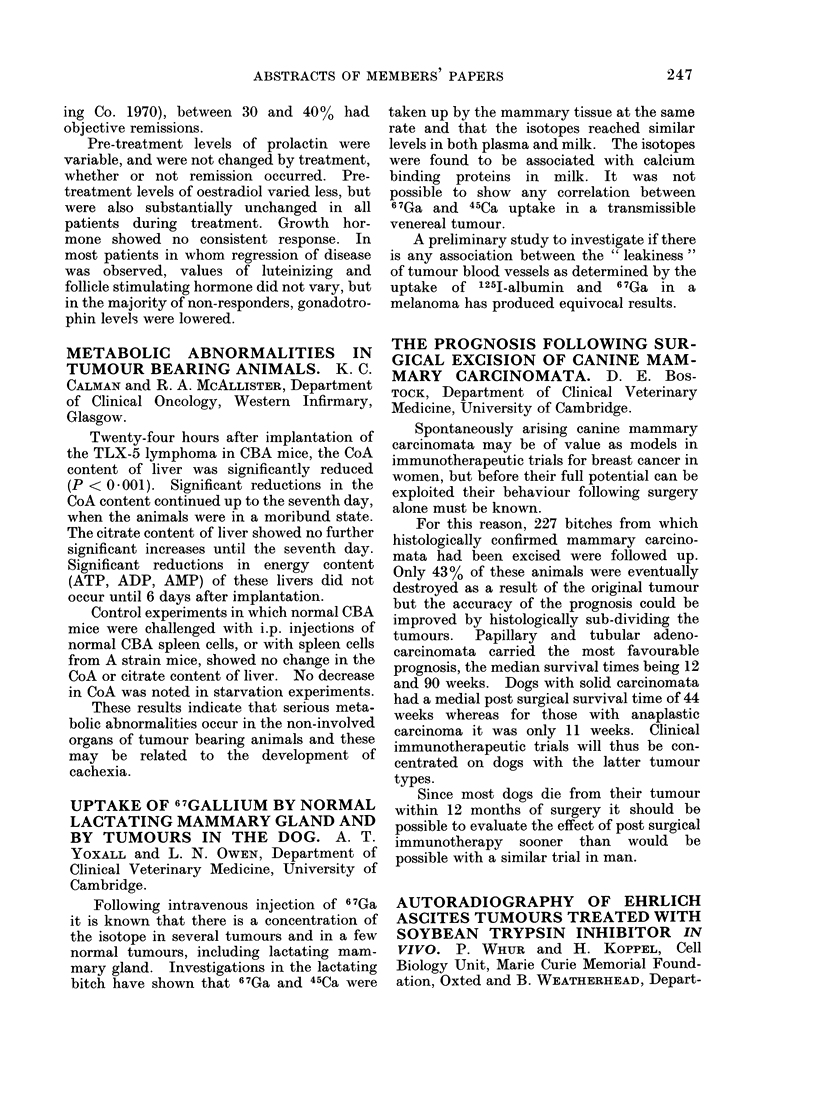# Proceedings: Uptake of 67gallium by normal lactating mammary gland and by tumours in the dog.

**DOI:** 10.1038/bjc.1975.178

**Published:** 1975-08

**Authors:** A. T. Yoxall, L. N. Owen


**UPTAKE OF $^{67}$GALLIUM BY NORMAL LACTATING MAMMARY GLAND AND BY TUMOURS IN THE DOG.** A. T. Yoxall and L. N. Owen, Department of Clinical Veterinary Medicine, University of Cambridge.

Following intravenous injection of $^{67}$Ga it is known that there is a concentration of the isotope in several tumours and in a few normal tumours, including lactating mammary gland. Investigations in the lactating bitch have shown that $^{67}$Ga and $^{45}$Ca were taken up by the mammary tissue at the same rate and that the isotopes reached similar levels in both plasma and milk. The isotopes were found to be associated with calcium binding proteins in milk. It was not possible to show any correlation between $^{67}$Ga and $^{45}$Ca uptake in a transmissible venereal tumour.

A preliminary study to investigate if there is any association between the "leakiness" of tumour blood vessels as determined by the uptake of $^{125}$I-albumin and $^{67}$Ga in a melanoma has produced equivocal results.